# CTLA4 Gene Polymorphisms Influence the Incidence of Infection after Renal Transplantation in Chinese Recipients

**DOI:** 10.1371/journal.pone.0070824

**Published:** 2013-08-27

**Authors:** Yifeng Guo, Fang Guo, Chongyang Wei, Jianxin Qiu, Yong Liu, Yu Fang, Junwei Gao

**Affiliations:** 1 Urological Center and Organ Transplantation Center, Shanghai First People's Hospital, Shanghai Jiao Tong University, Shanghai, China; 2 Lab of Tumor Targeted Therapy, Key Laboratory of Systems Biology, Chinese Academy of Sciences, Shanghai, China; 3 Department of Pharmacy, Shanghai First People's Hospital, Shanghai Jiao Tong University, Shanghai, China; Shanghai Jiao Tong University School of Medicine, China

## Abstract

**Background:**

Immunosuppressive therapy is usually administered following renal transplantation to protect the graft from rejection. However, this often causes complications such as infections to occur. Single nucleotide polymorphisms (SNPs) within the CTLA4 gene, such as −1772T/C (rs733618), +49A/G (rs231775) and +6230 G/A (rs3087243), can affect graft rejection and the long-term clinical outcome of organ transplantation. The role of CTLA4 SNPs in T cell-mediated immunity in renal transplantation and association with infection after transplantation is unknown.

**Methods:**

In this study, the risk of infection according to CTLA4 SNPs was investigated in 304 patients who received kidney graft transplants between 2008 and 2012.

**Results:**

The frequency of the rs4553808 GG genotype was significantly higher in recipients with viral infection (14.89%) than in those without infections (3.50%) (Bonferroni-adjusted p = 0.005). A significant difference (p = 0.001) in patients with the rs4553808 GG genotype from those with the AA+AG genotypes was found in the viral cohort using the log-rank test. A significant association was found between the rs4553808 genotype and onset of viral infection in transplant recipients (p = 0.001). The frequencies of the CGTAG and CGCAG haplotypes were significantly higher in the viral infection group (9.6% and 5.3%) than in the non-viral infection group (3.8% and 1.4%) (p = 0.0149 and p = 0.0111). No association between any CTLA4 SNP and bacterial infection was found. Multivariate analyses revealed that one risk factor, the use of antibody induction therapy (p = 0.007), was associated with bacterial infection, and two risk factors, antibody use (p = 0.015) and recipient rs4553808 genotype (p = 0.001), were associated with viral infection.

**Conclusions:**

The rs4553808 GG genotype may be a risk factor for viral infection in kidney transplantation. The CTLA4 haplotypes CGTAG and CGCAG were partially associated with the development of viral infection in Chinese kidney transplant recipients.

## Introduction

Immunosuppressive drugs, such as cyclosporine A (CsA), tacrolimus (TAC), mycophenolate mofetil (MMF) or prednisone (Pred), are typically administered to renal transplant patients to prevent graft rejection. However, this can lead to complications including various bacterial, viral, and fungal infections, such as pneumonia and urinary tract infections [1]–[5].

Cytotoxic T lymphocyte-associated antigen 4 (*CTLA4*) is a critical negative regulator of the T cell-mediated immune response and a key element that induces immune tolerance in the immune system [6]. It is also expressed constitutively on the surface of regulatory T cells (Tregs); it is detectable on approximately 50% of Tregs, but found on only <1% of naive helper T cells [7]. *CTLA4* ligation on Tregs results in a significant decrease in the presentation capacity of antigen-presenting cells and effector T cell downregulation in mice [8]. *CTLA4* plays an important role in the downregulation of the immune response. The expression pattern of the protein encoded by *CTLA4* was found to be altered by the polymorphisms rs4553808 (−1661A/G) and rs5742909 (−318C/T) in the *CTLA4* gene promoter [9]. Similarly, the rs733618 (−1772T) allele was found to decrease the transcription level of *CTLA4* by influencing the binding of transcription factors [10]. The rs231775 (+49A/G) SNP is located within the signal peptide of the molecule and influences the expression of the full-length isoform on the T cell surface. The rs3087243 (+6230G/A) SNP is located within the 3′ untranslated region of the *CTLA-4* gene and was found to be associated with susceptibility to autoimmune diseases [11]. Furthermore, the SNPs within the *CTLA4* gene, such as −1772T/C (rs733618), +49A/G (rs231775) and +6230 G/A (rs3087243), play an influential role in graft rejection and the long-term clinical outcome of organ transplantation [12]–[17].

The *CTLA4* gene polymorphism has been suggested to influence infection after pediatric heart transplantation. Ohmann et al. [18] reported that SNP *CTLA4* +49(rs231775) may be associated with late post-transplantation viral infection in pediatric heart transplant recipients in the USA.

The role of *CTLA4* SNPs in T cell-mediated immunity in renal transplantation and association with infection after transplantation is unknown. Therefore, this study was designed to investigate the associations between five *CTLA4* SNPs (rs733618 C/T, rs4553808 A/G, rs5742909 C/T, rs231775 A/G, and rs3087243 G/A) and infection in Chinese kidney transplant recipients.

## Materials and Methods

### Patients

This study included 304 transplant recipients (264 deceased donor cases and 40 living donor cases; 192 men and 112 women) in the Shanghai Organ Transplantation Center between Jan 2008 and Dec 2012; post-transplant infections occurred in 123 recipients, and no infection occurred in 181 cases. Of the 123 patients with infections, 61 recipients suffered from bacterial infection, 47 from viral infection, 11 from fungal infection and 4 from other infection. The mean age of the patients in the study was 43.76±12.01 years. Overall, 287 of the patients received transplantation for chronic glomerulonephritis, 9 for polycystic kidney disease, and 8 for pyelonephritis. Only subjects with negative infection status were eligible for transplantation. All of the recipients were blood group-matched with their donors and were tested for panel-reactive antibody and HLA-A-B-DR matching.

The study protocol was approved by the Ethics Committee review boards of our hospital and Shanghai Jiao Tong University, and informed consent was obtained from all patients. Each organ donation or transplant in our center was strictly selected according to the guidelines of the Ethics Committee review board of our hospital, the Organ Transplant Regulation Committee of Shanghai Jiao Tong University and the Declaration of Helsinki. The research process, including the collection and storage of blood, isolation of DNA and determination of gene polymorphisms, was explained in detail to every candidate patient. Every participant provided written informed consent.

Infection was defined as clinical evidence of an infectious process occurring within the first year after transplantation. The date of infection, type of infection (bacterial, viral, fungal, protozoan, or no organism identified), location of infection, therapy administered, and outcome were reported on standardized forms.

In our hospital, the virus was detected by Fluorescent PCR (detection of viral DNA) and immunohistory (detection of viral antibody and antigen). Viral DNA detection was used in cytomegalovirus (CMV), BK virus, Epste in-Barr virus (EBV), Human papilloma virus (HPV), Herpes simplex virus (HSV) and hepatitis B virus (HBV). Viral antibody and/or antigen detection was used in hepatitis C virus (HCV), CMV, HBV, EBV, human parvo-virus and HSV. For the detection of bacteria (Including Gram-positive bacteria and gram-negative bacteria), smear and culture were applied in sputum, blood, urine and bronchoalveolar lavage fluid.

This was a cross-sectional study. Each patient underwent a 12-month follow-up observation, and clinical information was gathered from clinical observations, medical records and outpatient follow-up visits. Severe patients required hospitalization and intravenous antimicrobial therapy. Patients were monitored monthly, and additional data collection forms were completed to record any significant clinical events, including rejection, severe infection and death. Demographic characteristics (gender and age at transplantation), transplant etiology, HLA match, and clinical outcomes were collected prospectively. The exclusion criteria were (1) observation time less than 12 months, (2) death for other reasons (except for various infections) within 12 months post-transplantation, (3) discontinued use of immune suppressants due to graft function failure, and (4) inability to provide written informed consent. This study was performed from Jan 2008 to Dec 2012. All patients were discharged within approximately 30 days after surgery and visited our outpatient clinics thereafter; all patients who did not meet the exclusion criteria (n = 304) were invited to take part in the present study.

### Immunosuppression

Mycophenolate mofetil (MMF) 1.0 was given as a premedicant. A subset of recipients used antibodies for induction therapy, including multiclonal antibodies (ATG and ALG) and monoclonal antibodies (basiliximab and daclizumab). Intravenous infusion of 500 mg/d methylprednisolone was applied throughout the procedure and for 2 days after the operation. The dose was then decreased to 360 mg, 180 mg, 80 mg and 40 mg on each subsequent day, followed by the initiation of prednisone (15–20 mg/d) as a maintenance therapy. Triple therapy with cyclosporine A (CsA)/tacrolimus (TAC), MMF and prednisone was administered beginning on the third day after the operation. The dosage of MMF was 1.0–1.5 g/d, with a weight of 60 kg as the critical value. CsA and TAC were given at initial doses of 8 mg/kg/d and 0.2 mg/kg/d, respectively, and then adjusted according to the plasma drug concentrations and the serum creatinine concentrations. Acute rejection was diagnosed based on clinical and biopsy findings using the Banff 97 working classification for renal allograft pathology (modified) [19] as the pathological rejection criteria.

### Sample collection and polymorphism genotyping

A total of 304 patients were included in this study. Peripheral blood samples (3 ml) were collected, the DNA was extracted, and the SNPs of *CTLA4* were genotyped using polymerase chain reaction (PCR) and direct sequencing. The primers and annealing temperatures (ATs) employed for rs733618 C/T, rs4553808 A/G, rs5742909 C/T, rs231775 A/G and rs3087243 G/A are shown in Table S1.

### Statistical analysis

Comparisons of clinical characteristics between patients with and without infection were analyzed by the Pearson *χ^2^* test and an independent sample test. We assessed the Hardy–Weinberg equilibrium (HWE) for both infection and non-infection using the *χ^2^* test. Haploview version 4.2 software was used to calculate linkage disequilibrium (LD) [20]. A correlation test was used to assay the degree of correlation between the onset of infection and the *CTLA4* genotypes of the recipients. Genotype associations were analyzed using a dominant model (minor-allele homozygotes plus heterozygotes vs. major-allele homozygotes) and a recessive model (minor-allele homozygotes vs. heterozygotes plus major-allele homozygotes). The allelic frequencies were counted in a single strand of measured DNA. The differences in the genotype distributions between groups were analyzed by the *χ^2^* test or Fisher's exact test. The time to infection was designated as the number of days post-transplantation of the first infection. The associations between the *CTLA4* SNPs and time (days) to first infection were analyzed with the Kaplan-Meier test. Multivariate logistic regression analyses were used to evaluate several risk factors, including age, gender, primary disease, number of HLA mismatches, acute rejection, blood transfusion, *CTLA4* SNPs and antibody induction therapy. These risk factors were analyzed together. We explored the haplotype associations for 5 SNPs using Haploview version 4.2. All statistical tests were two-sided, and statistical significance was set at *p*<0.05. Correction for multiple testing was carried out using the Bonferroni method. Statistical analysis was performed with SPSS (Statistical Package for the Social Sciences) version 11.5 software (SPSS Inc., Chicago IL, USA).

## Results

### Baseline characteristics of 304 renal transplant recipients

A total of 304 patients were recruited to the study, including 192 male and 112 female cases. During the first 12 months post-transplantation, a total of 20.07% (61/304) recipients developed bacterial infection, 15.46% (47/304) developed viral infection, 3.62% (11/304) developed fungal infection and 1.32% (4/304) developed another infection. The baseline characteristics of the 304 renal transplant recipients and types of infection are listed in Table 1. No significant differences in age, sex, primary disease, human leukocyte antigen mismatches, blood transfusion, renal transplantation or immunosuppressant regimen were found between patients with infection and those without (controls) (Table 1). The incidence of acute rejection (AR) following renal transplantation was similar in both groups. However, the groups that suffered from bacterial infection and from viral infection were significantly different from the control (non-infection group) (*p* = 0.007 and *p* = 0.015).

**Table 1:** Comparison of clinical characteristics between patients with infection and non-infection.

Characteristics	Bacterial infection (n = )61(%)	Non-bacterial infection (n = 243)(%)	*p* value	Viral infection (n = 47)(%)	Non-viral infection (n = 257)(%)	*p* value
Mean age±SD	44.59±11.07	43.55±12.24	0.547	42.13±13.93	44.06±11.63	0.312
Sex			0.888			0.446
Male/Female	39/22	153/90		32/15	160/97	
Primary diseases			0.750			0.830
Chronic glomerulonephritis	58(95.08)	229(94.24)		44(93.62)	243(94.56)	
Polycystic kidney	1(1.63)	8(3.29)		2(4.26)	7(2.72)	
Pyelonephophritis	2(3.27)	6(2.47)		1(2.13)	7(2.72)	
Number of HLA -mismatch	2.49±−0.87	2.45±0.85	0.728	2.40±0.83	2.47±0.88	0.649
Real transplantation						
Lived/cadaver	7/54	33/210	0.665	8/39	32/225	0.396
Immunosuppressive regiment			0.920			0.099
CsA+MMF+Pred	40	161		36	165	
TAC+MMF+Pred	21	82		11	92	
Blood transfusion	10/50	50/193	0.463	8/39	52/205	0.611
Antibody induction			0.007			0.015
Using antibody	19(31.15)	39(16.05)		15(31.91)	43(16.73)	
Non-using antibody	42(68.85)	204(83.95)		32(68.09)	214(83.27)	
Rejection			0.058			0.838
AR	32(52.46)	95(39.09)		19(40.43)	108(42.02)	
Non-AR	29(47.54)	148(60.91)		28(59.57)	149(57.98)	

CsA: cyclosporine, MMF: mycophenolate mofetil, Pred: prednisone, TAC: tacolimous,

AR: acute rejection, non-AR: non-acute rejection, DILI: drug induced liver injury.

### Associations between the CTLA4 SNPS and two types of infection

All polymorphisms were in Hardy-Weinberg equilibrium. Using Haploview version 4.2 software, the five loci were found to be in linkage disequilibrium (LD) (D′ = 0.900–1.000). No significant differences in the genotype distributions of rs733618, rs4553808, rs5742909, rs231775 or rs3087243 were found between patients with bacterial infection and those without bacterial infection (Table 2). In the viral infection group, no significant differences were found among rs733618, rs5742909, rs231775 and rs3087243; however, the frequency of the rs4553808 GG genotype in recipients with viral infection was significantly higher (14.89%) than in those recipients without infection (3.50%) (*p* = 0.001, OR = 4.822, 95% CI = 1.700–13.679, Bonferroni-adjusted *p* = 0.005) (Table 3). No differences in the determined allelic frequencies of rs733618, rs4553808, rs5742909, rs231775 or rs3087243 were found between the bacterial infection cohort and the viral infection cohort (Table 4 and Table 5).

**Table 2:** The genotype distribution of the *CTLA4* polymorphisms in patients with bacterial infection and non-bacterial infection.

Locus	Genotype	Patients with bacterial infection(n = 61)n(%)	Patients with non-bacterial infection(n = 243)n(%)	Model	OR (95% CI)	*p* value
rs733618	TT	22(36.07)	90(37.04)	Dominant	1.043(0.582–1.870)	0.888
	CT	29(47.54)	119(48.97)	Recessive	1.205(0.559–2.600)	0.634
	CC	10(16.39)	34(13.99)			
rs4553808	AA	38(62.30)	158(65.02)	Dominant	1.125(0.629–2.012)	0.691
	AG	20(32.79)	72(29.63)	Recessive	0.915(0.252–3.318)	1.000*
	GG	3(4.92)	13(5.35)			
rs5742909	CC	39(63.93)	165(67.90)	Dominant	1.193(0.663–2.148)	0.555
	CT	21(34.43)	71(29.22)	Recessive	0.562(0.068–4.655)	1.000*
	TT	1(1.64)	7(2.88)			
rs231775	GG	23(37.70)	97(39.91)	Dominant	1.098(0.616–1.956)	0.752
	AG	29(47.54)	115(47.33)	Recessive	1.184(0.531–2.639)	0.680
	AA	9(14.75)	31(12.76)			
rs3087243	GG	46(75.40)	182(74.88)	Dominant	0.973(0.507–1.865)	0.934
	AG	13(21.31)	47(19.34)	Recessive	0.554(0.123–2.507)	0.748*
	AA	2(3.28)	14(5.76)			

* Fisher's Exact Test;

OR: odds ratio, CI: confidence intervals.

**Table 3:** The genotype distribution of the *CTLA4* polymorphisms in patients with viral infection and non-viral infection.

Locus	Genotype	Patients with viral infection(n = 47)N(%)	Patients with non-viral infection(n = 257)N(%)	Model	OR (95% CI)	*p* value
rs733618	TT	20(42.55)	92(35.80)	Dominant	0.753(0.400–1.416)	0.377
	CT	19(40.43)	129(50.19)	Recessive	1.259(0.545–2.912)	0.589
	CC	8(17.02)	36(14.01)			
rs4553808	AA	30(63.83)	166(64.59)	Dominant	1.034(0.541–1.975)	0.920
	AG	10(21.28)	82(31.91)	Recessive	4.822(1.700–13.679)	0.001*
	GG	7(14.89)	9(3.50)			
rs5742909	CC	30(63.83)	174(67.71)	Dominant	1.188(0.620–2.275)	0.603
	CT	15(31.91)	77(29.96)	Recessive	1.859(0.364–9.503)	0.358*
	TT	2(4.26)	6(2.33)			
rs231775	GG	21(44.68)	99(38.52)	Dominant	0.776(0.414–1.453)	0.427
	AG	18(38.30)	126(49.03)	Recessive	1.442(0.619–3.361)	0.394
	AA	8(17.02)	32(12.45)			
rs3087243	GG	39(82.98)	189(73.54)	Dominant	0.570(0.254–1.281)	0.202
	AG	6(12.77)	54(21.01)	Recessive	0.771(0.170–3.511)	1.000*
	AA	2(4.25)	14(5.45)			

* Fisher's Exact Test;

OR: odds ratio, CI: confidence intervals.

**Table 4:** The allele distribution of *CTLA4* polymorphisms in patients with bacterial infection and non-bacterial infection.

Locus	Allele	Patients with bacterial infection(n = 122)n(%)	Patients with non-bacterial infection(n = 486)n(%)	OR (95% CI)	*p* value
rs733618	T	73(59.84)	299(61.52)	0.932(0.621–1.398)	0.733
	C	49(40.16)	187(38.48)		
rs4553808	A	96(78.69)	388(79.84)	0.933(0.573–1.517)	0.779
	G	26(21.31)	98(20.16)		
rs5742909	C	99(81.15)	401(82.51)	0.912(0.548–1.520)	0.725
	T	23(18.85)	85(17.49)		
rs231775	G	75(61.48)	309(63.58)	0.914(0.607–1.376)	0.667
	A	47(38.52)	177(36.42)		
rs3087243	G	105(86.07)	411(84.57)	1.127(0.638–1.990)	0.680
	A	17(13.93)	75(15.43)		

OR: odds ratio, CI: confidence intervals.

**Table 5:** The allele distribution of *CTLA4* polymorphisms in patients with viral infection and non-viral infection.

Locus	Allele	Patients with viral infection(n = 94)n(%)	Patients with non- viral infection(n = 514)n(%)	OR (95% CI)	*p* value
rs733618	T	59(62.77)	313(60.89)	1.083(0.687–1.705)	0.732
	C	35(37.23)	201(39.11)		
rs4553808	A	70(74.47)	414(80.54)	0.705(0.422–1.176)	0.179
	G	24(25.53)	100(19.46)		
rs5742909	C	75(79.79)	425(82.68)	0.827(0.476–1.437)	0.499
	T	19(20.21)	89(17.32)		
rs231775	G	60(63.83)	324(63.04)	1.035(0.655–1.635)	0.883
	A	34(36.17)	190(36.96)		
rs3087243	G	84(89.36)	432(84.05)	1.594(0.794–3.201)	0.186
	A	10(10.64)	82(15.95)		

OR: odds ratio, CI: confidence intervals.

Kaplan-Meier analysis was used to examine the relationships between *CTLA4* SNPs and bacterial or viral infection (Table S2 and Table S3); no significant differences in the genotype frequencies of rs733618, rs4553808, rs5742909, rs231775 or rs3087243 existed between the bacterial infection and non-bacterial infection groups (Figure S1). A significant difference (*p* = 0.001) was found between patients with the rs4553808 GG genotype and those with the AA+AG genotypes using the log-rank test (Figure S2) in the viral cohort. The mean and 95% CI of time to infection for the GG and AG+AA groups were 298.188±24.555 (95% CI: 250.059–346.316) days and 340.736±4.155 (95% CI: 332.593–348.880) days, respectively. A significant association between the rs4553808 genotype and onset of viral infection was found (*p* = 0.001).

To further examine the associations of infection with these variables, univariate and multivariate analyses were carried out for the following variables: age, gender, primary disease, immunosuppressive regimen, blood transfusion, HLA mismatch, renal transplantation, antibody induction therapy, acute rejection (AR) and rs4553808 genotype (Table 6).

**Table 6:** Association between bacterial or viral infection and several risk factors.

Variables	Multivariable analysis					
	Bacterial infection (n = 61)(%)	Non-bacterial infection (n = 243)(%)	*p* value	Viral infection (n = 47)(%)	Non-viral infection (n = 257)(%)	*p* value
Mean age±SD	44.59±11.07	43.55±12.24	0.600	42.13±13.93	44.06±11.63	0.525
Sex			0.889			0.448
Male/Female	22/39	90/153		32/15	160/97	
Primary diseases			0.995			0.953
Chronic glomerulonephritis	58(95.08)	229(94.24)		44(93.62)	243(94.56)	
Polycystic kidney	1(1.63)	8(3.29)		2(4.26)	7(2.72)	
Pyelonephophritis	2(3.27)	6(2.47)		1(2.13)	7(2.72)	
Number of HLA -mismatch	2.49±−0.87	2.45±0.85	0.728	2.40±0.83	2.47±0.88	0.649
Real transplantation						
Lived/cadaver	7/54	33/210	0.665	8/39	32/225	0.396
Immunosuppressive regiment			0.920			0.099
CsA+MMF+Pred	40	161		36	165	
TAC+MMF+Pred	21	82		11	92	
Blood transfusion	10/50	50/193	0.465	8/39	52/205	0.612
Antibody induction			0.007			0.015
Using antibody	19(31.15)	39(16.05)		15(31.91)	43(16.73)	
Non-using antibody	42(68.85)	204(83.95)		32(68.09)	214(83.27)	
Rejection			0.059			0.839
AR	32(52.46)	95(39.09)		19(40.43)	108(42.02)	
Non-AR	29(47.54)	148(60.91)		28(59.57)	149(57.98)	
rs4553808 SNPs			0.893			0.001
GG	3(4.92)	13(5.35)		7(14.89)	9(3.50)	
AG+AA	58(95.08)	230(94.65)		40(85.11)	248(96.50)	

CsA: cyclosporine, MMF: mycophenolate mofetil, Pred: prednisone, TAC: tacolimous,

AR: acute rejection, non-AR: non-acute rejection.

Multivariate analyses revealed that age, gender, primary disease, immunosuppressive regimen, blood transfusion, HLA mismatch and renal transplantation were independent of bacterial and viral infection. However, the analyses showed that a risk factor, the use of antibody induction therapy (*p* = 0.007) was associated with bacterial infection. Two risk factors, the use of antibody induction therapy (*p* = 0.015) and the rs4553808 genotype (*p* = 0.001) were associated with viral infection.

### The association of CTLA4 haplotype and infection

No differences in the frequencies of nine haplotypes covering the 5 SNPs was observed between the bacterial infection and non-bacterial infection groups (Table 7). The frequencies of haplotypes CGTAG and CGCAG were significantly higher in the viral infection group (9.6% and 5.3%) than in the non-viral infection group (3.8% and 1.4%) (*p* = 0.0149 and *p* = 0.0111). No statistically significant differences between the viral infection and non-viral infection groups were found for any of the remaining haplotypes (*p*>0.05) (Table 8).

**Table 7:** The distribution of haplotypes in 5 locus of CTLA-4 between bacterial infection and non- bacterial infection.

Haplotype	Frequency (%)		*χ^2^*	*p* value
	Bacterial infection	Non-bacterial infection		
5 locus				
TACGG	56.4	57.3	0.029	0.8647
CACAG	15.5	15.8	0.006	0.9404
CGTAA	12.1	12.4	0.007	0.9350
CGTAG	5.9	4.5	0.441	0.5065
TACGA	0.9	2.4	0.990	0.3198
CACGG	2.5	1.9	0.180	0.6715
CGCAG	2.5	1.9	0.187	0.6656
TACAG	1.7	1.3	0.117	0.7321
CGCGG	0.8	1.4	0.289	0.5907

**Table 8:** The distribution of haplotypes in 5 locus of CTLA-4 between viral infection and non-viral infection.

Haplotype	Frequency (%)		*χ^2^*	*p* value
	Viral infection	Non-viral infection		
5 locus				
TACGG	62.6	56.1	1.403	0.2362
CACAG	10.6	16.7	2.208	0.1373
CGTAA	10.5	12.7	0.337	0.5617
CGTAG	9.6	3.8	5.934	0.0149
TACGA	0.1	2.5	2.201	0.1379
CACGG	1.1	2.2	0.475	0.4909
CGCAG	5.3	1.4	6.444	0.0111
TACAG	0	1.6	1.459	0.2271
CGCGG	0	1.6	1.483	0.2234

## Discussion

In kidney transplant recipients, immunosuppressive therapy is usually administered as a triple regimen, typically including cyclosporine A (CsA)/tacrolimus (TAC)+mycophenolate mofetil (MMF)+prednisone (Pred). Antibody induction therapy is used in some recipients in cases of acute rejection. Antibody induction is favored because it produces a more effective immunosuppression and prolongs graft survival. However, various infections after surgery are common due to the use of immune suppression.

In Chinese Han populations, patients carrying *CTLA4*+49(rs231775) AA and A exhibited a higher frequency of chronic HBV infection, and haplotype+49A-318C was significantly over-represented [21]. Data indicated that the +49A allele increases the risk of development of viral and parasitic diseases (e.g., dengue, Chagas disease and American cutaneous leishmaniasis) but confers resistance to autoimmune diseases (MG, PE) [22]. Ohmann et al. [18] reported that SNP *CTLA4* +49(rs231775) may be associated with late post-transplantation viral infection in pediatric heart recipients in the USA. For the SNP rs5742909 C/T, several studies have shown that the CC genotype is associated with viral disease and chronic HBV infection [23]. However, no statistically significant associations for the SNPs rs5742909 and rs231775 were found in the study. This lack of association may be due to the sample size and lack of power to detect an association; furthermore, the frequency of the G allele at the *CTLA4* +49 (rs231775) locus is much higher in the Chinese population than in other populations [24]. This may indicate that this genetic bias does not play a significant role in infection susceptibility. A recent study suggested that the *CTLA4*+49(rs231775)GG genotype was also associated with increased interferon-γ production after immune stimulation [25].

The loci rs733618 (−1772) and rs4553808(−1661), rs3087243 (+6230) have been studied in relation to type 1 diabetes, systemic lupus erythematosus (SLE), cancer, organ transplantation and other diseases [17], [26]–[30]. It has been speculated that rs4553808 (−1661) AA plays a protective role in the autoimmune disease and that the genotype GG as the risk factor for SLE. Likewise, rs733618 (−1772) TT was found to be associated with the risk of AR following renal transplantation in our previous study [17]. Our study revealed that the frequency of the rs4553808 GG genotype was higher in the viral infection cohort than in the non-viral infection group (*p* = 0.001). The statistical significance remained after correction for multiple testing (correction *p* = 0.005). It is likely that the rs4553808 (−1661) G allele affects *CTLA4* transcription by altering a putative transcription factor binding site as rs5742909 (−318) C/T upregulates *CTLA4* transcription, resulting in the abnormal expression of *CTLA4* [31].

The frequencies of the haplotypes CGTAG and CGCAG, including the rs231775 A allele, were significantly higher in the viral infection group than in the non-viral infection group (*p* = 0.0149 and *p* = 0.0111).

Susceptibility factors such as age and gender are thought to confer an increased risk for the development of infectious complications [32]. Our multivariate analysis showed that age and gender are not apparently susceptibility factors for the development of infection (*p* = 0.250 and *p* = 0.448, respectively), which contradicts some previous studies. In general, increased age is a risk factor for infection following transplantation [32]. Women are widely viewed as being more likely to develop infections [33].

The χ^2^ test showed no correlation between bacterial or viral infection and acute rejection (AR) (*p* = 0.059 and *p* = 0.839) (Table 7). The percentage of AR recipient with bacterial infection (32/61, 52.46%) was higher than that of non-AR recipients (95/243, 39.09%), although this difference only approached significance (*p* = 0.058). In our previous study [17], a correlation between *CTLA4* SNPs and AR was observed. That data was not sufficient to determine whether AR is associated with a higher risk of infection, but a multivariate analysis published by Mourad et al. [33] this year showed that acute rejection was a risk factor for infectious complications after renal transplantation. The association between AR and infection in that study is statistically weak, with a *p* value close to the threshold. It is possible that different populations and other statistical biases could affect these results. Therefore, further studies in different populations and larger patient cohorts are needed to address this question. Patients with biopsy-confirmed AR typically receive an intravenous steroid bolus and antibodies, which may to some extent precipitate the development of infection.

Multivariate analysis showed that antibody use was a risk factor (*p* = 0.007) for bacterial infection and that antibody use (*p* = 0.015) and recipient rs4553808 genotype (*p* = 0.001) were risk factors associated with viral infection. Susceptibility factors such as antibody induction therapy may confer an increased risk of infection. Baek et al. [34] discovered that serious infectious complications increased in kidney transplant recipients who were treated with an anti-CD20 antibody (Rituximab).

In conclusion, the *CTLA4* haplotypes CGTAG and CGCAG were partially associated with the development of viral infection in Chinese kidney transplant recipients. The rs4553808 GG genotype may be a risk factor for viral infection in kidney transplantation. The role of *CTLA4* SNPs in infection following renal transplantation has not been completely elucidated and needs to be studied in greater depth.

**Patient consent:** Obtained.

**Provenance and peer review:** Not commissioned; externally peer reviewed.

## Supporting Information

Figure S1 **Association between** ***CTLA4*** **SNPs and early onset of bacterial infection in renal transplantation.** No statistical differences for rs733618 (A), rs4553808 (B), rs5742909 (C), rs231775 (D) or rs3087243(E) were found between bacterial infection and non-bacterial infection.
(TIF)

Figure S2 **Association between** ***CTLA4*** **SNPs and early onset of viral infection in renal transplantation.** No statistical differences for rs733618 (A), rs5742909 (C), rs231775 (D) or rs3087243(E) were found between viral infection and non-viral infection. A significant difference (p = 0.001) was found between patients bearing the rs4553808 GG genotype and those with the AA+AG genotypes using the log-rank test (B).
(TIF)

Table S1 **PCR primers of the CTLA4 SNP used in the study.**
(DOC)

Table S2 **Correlation between onset of bacterial infection and CTLA4 genotypes in recipients.**
(DOC)

Table S3 **Correlation between onset of viral infection and CTLA4 genotypes in recipients.**
(DOC)
