# Supplementary figures and images for: CTLA4 Gene Polymorphisms Influence the Incidence of Infection after Renal Transplantation in Chinese Recipients
Source: PLoS One. 2013 Aug 27;8(8):e70824. doi: 10.1371/journal.pone.0070824 (PMC3754976; doi:10.1371/journal.pone.0070824)

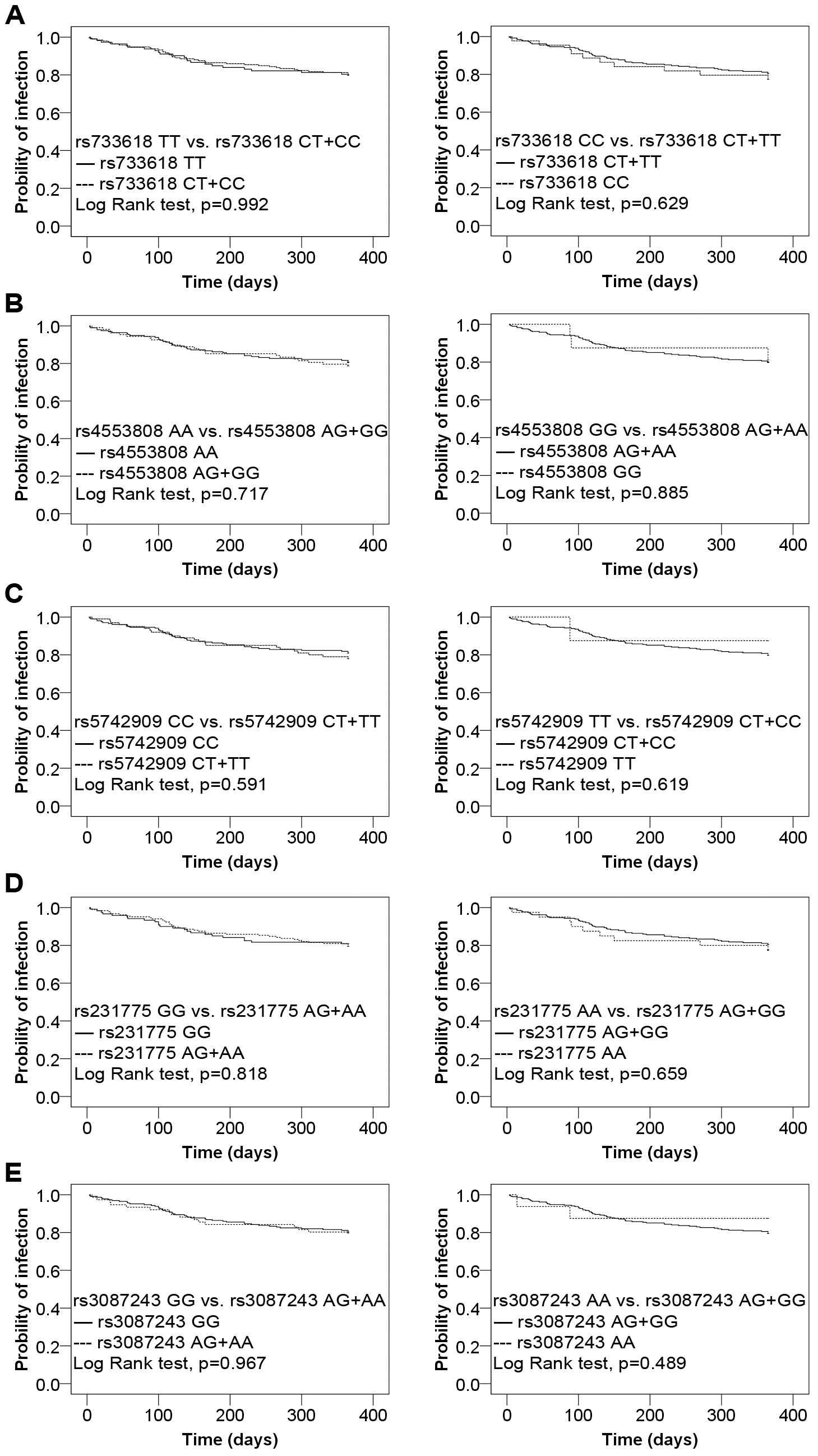

Supplement: Figure S1 — Association between CTLA4 SNPs and early onset of bacterial infection in renal transplantation. No statistical differences for rs733618 (A), rs4553808 (B), rs5742909 (C), rs231775 (D) or rs3087243(E) were found between bacterial infection and non-bacterial infection. (TIF) [file pone.0070824.s001.tif]

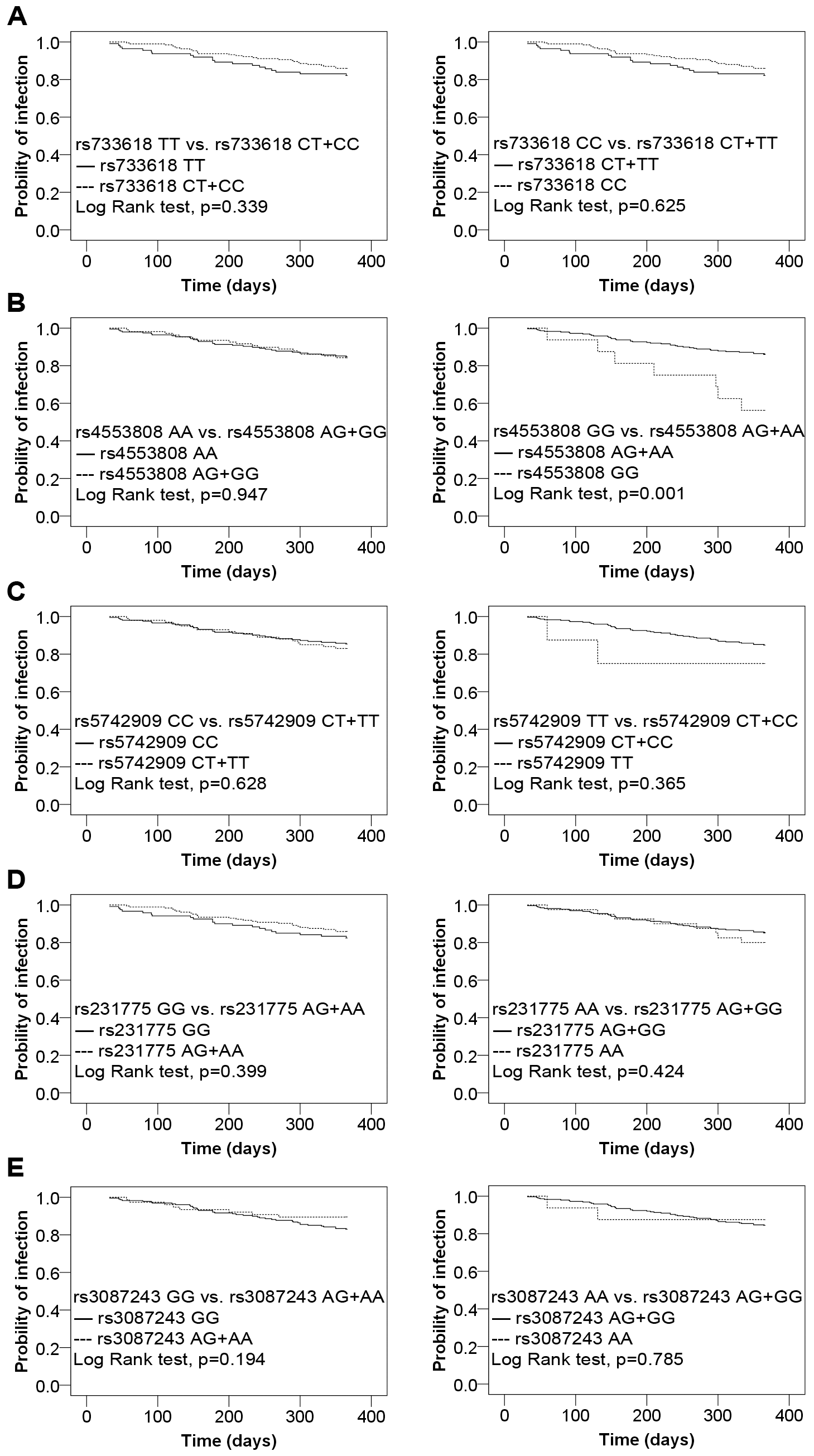

Supplement: Figure S2 — Association between CTLA4 SNPs and early onset of viral infection in renal transplantation. No statistical differences for rs733618 (A), rs5742909 (C), rs231775 (D) or rs3087243(E) were found between viral infection and non-viral infection. A significant difference (p = 0.001) was found between patients bearing the rs4553808 GG genotype and those with the AA+AG genotypes using the log-rank test (B). (TIF) [file pone.0070824.s002.tif]
