# Supplementary material for: CTLA4 Gene Polymorphisms Influence the Incidence of Infection after Renal Transplantation in Chinese Recipients
Source: PLoS One. 2013 Aug 27;8(8):e70824. doi: 10.1371/journal.pone.0070824 (PMC3754976; doi:10.1371/journal.pone.0070824)
Supplement: Table S1 — PCR primers of the CTLA4 SNP used in the study. (DOC) [file pone.0070824.s003.doc]

**Table S1** PCR primers of the CTLA4 SNP used in the study

| **Locus** | **AT (°C)** | **Primer pairs (5’→ 3’)** | **Amplicon size (bp)** |
| --- | --- | --- | --- |
| rs733618, rs4553808 | 58 | CTAAGAGCATCCGCTTGCACCT | 486 |
|  |  | TTGGTGTGATGCACAGAAGCCTTTT |  |
| rs5742909 | 56 | AAATGAATTGGACTGGATGGT | 226 |
|  |  | TTACGAGAAAGGAAGCCGTG |  |
| rs231775 | 58 | GCTCTACTTCCTGAAGACCT | 162 |
|  |  | AGTCTCACTCACCTTTGCAG |  |
| rs3087243 | 59 | AGGAAGGCAGATCAAAATGC | 202 |
|  |  | CACCACTATTTGGGATATAACA |  |
