# Supplementary material for: CTLA4 Gene Polymorphisms Influence the Incidence of Infection after Renal Transplantation in Chinese Recipients
Source: PLoS One. 2013 Aug 27;8(8):e70824. doi: 10.1371/journal.pone.0070824 (PMC3754976; doi:10.1371/journal.pone.0070824)
Supplement: Table S2 — Correlation between onset of bacterial infection and CTLA4 genotypes in recipients. (DOC) [file pone.0070824.s004.doc]

**Table S2** Correlation between onset of bacterial infection and *CTLA4* genotypes in recipients

| **Locus** | **Genotype** | **Patients with bacterial infection (n=61)(%)** | **Patients with non-bacterial infection (n=243)(%)** | **Total counts (n=304)** | **Means (days)** | **95% CI** | ***p* value*** |
| --- | --- | --- | --- | --- | --- | --- | --- |
| rs733618 | TT | 22(36.07) | 90(37.04) | 112 | 319.018±9.536 | 300.328-337.708 | 0.922 |
|  | CT +CC | 39(63.93) | 153(62.96) | 192 | 322.229±7.151 | 308.213-336.245 |  |
|  | CC | 10(16.39) | 34(13.99) | 44 | 315.477±16.486 | 283.164-347.791 | 0.629 |
|  | CT + TT | 51(83.61) | 209(86.01) | 260 | 321.988±6.145 | 309.944-334.033 |  |
| rs4553808 | AA | 38(62.30) | 158(65.02) | 196 | 321.276±7.172 | 307.219-335.332 | 0.717 |
|  | AG+GG | 23(37.70) | 85(34.98) | 108 | 320.630±9.721 | 301.577-339.683 |  |
|  | GG | 3(4.92) | 13(5.35) | 16 | 330.500±27.948 | 275.721-385.279 | 0.885 |
|  | AG+ AA | 58(95.08) | 230(94.65) | 288 | 320.521±5.906 | 308.945-332.097 |  |
| rs5742909 | TT | 1(1.64) | 7(2.88) | 8 | 330.375±32.389 | 266.893-393.857 | 0.619 |
|  | CT +CC | 60(98.36) | 236(97.12) | 296 | 320.794±5.812 | 309.403-332.185 |  |
|  | CC | 39(63.93) | 165(67.90) | 204 | 321.436±7.049 | 307.620-335.252 | 0.591 |
|  | CT + TT | 22(36.07) | 78(32.10) | 100 | 320.250±10.045 | 300.562-339.938 |  |
| rs231775 | GG | 23(37.70) | 97(39.92) | 120 | 318.233±9.386 | 299.837-336.630 | 0.818 |
|  | AG+AA | 38(62.30) | 146(60.08) | 184 | 322.880±7.198 | 308.772-336.988 |  |
|  | AA | 9(14.75) | 31(12.76) | 40 | 314.150±17.927 | 279.014-349.286 | 0.659 |
|  | AG+ GG | 52(85.25) | 212(87.24) | 264 | 322.091±6.070 | 310.193-333.989 |  |
| rs3087243 | GG | 46(75.41) | 182(74.90) | 228 | 321.820±6.537 | 309.009-334.632 | 0.967 |
|  | AG+AA | 15(24.59) | 61(25.10) | 76 | 318.724±11.804 | 295.589-341.859 |  |
|  | AA | 2(3.28) | 14(5.76) | 16 | 325.750±26.167 | 274.463-377.037 | 0.489 |
|  | AG+ GG | 59(96.72) | 229(94.24) | 288 | 320.785±5.863 | 309.294-322.275 |  |

CI: confidence intervals, *log-rank test
