# Supplementary material for: CTLA4 Gene Polymorphisms Influence the Incidence of Infection after Renal Transplantation in Chinese Recipients
Source: PLoS One. 2013 Aug 27;8(8):e70824. doi: 10.1371/journal.pone.0070824 (PMC3754976; doi:10.1371/journal.pone.0070824)
Supplement: Table S3 — Correlation between onset of viral infection and CTLA4 genotypes in recipients. (DOC) [file pone.0070824.s005.doc]

**Table S3** Correlation between onset of viral infection and *CTLA4* genotypes in recipients

| **Locus** | **Genotype** | **Patients with viral infection (n=47)(%)** | **Patients with non- viral infection (n=257)(%)** | **Total counts (n=304)** | **Means (days)** | **95% CI** | ***p* value*** |
| --- | --- | --- | --- | --- | --- | --- | --- |
| rs733618 | TT | 20(42.55) | 92(35.80) | 112 | 330.080±8.211 | 313.986-346.175 | 0.339 |
|  | CT +CC | 27(57.45) | 165(64.20) | 192 | 343.406±4.546 | 334.497-352.316 |  |
|  | CC | 8(17.02) | 36(14.01) | 44 | 338.545±10.265 | 318.425-358.666 | 0.625 |
|  | CT + TT | 39(82.98) | 221(85.99) | 260 | 338.488±4.567 | 329.537-347.440 |  |
| rs4553808 | AA | 30(63.83) | 166(64.59) | 196 | 337.663±5.387 | 327.104-348.222 | 0.947 |
|  | AG+GG | 17(36.17) | 91(35.41) | 108 | 340.009±6.545 | 327.181-352.838 |  |
|  | GG | 7(14.89) | 9(3.50) | 16 | 298.188±24.555 | 250.059-346.316 | 0.001 |
|  | AG+ AA | 40(85.11) | 248(96.50) | 288 | 340.736±4.155 | 332.593-348.880 |  |
| rs5742909 | TT | 2(4.26) | 6(2.33) | 8 | 297.625±41.733 | 215.828-379.422 | 0.365 |
|  | CT +CC | 45(95.74) | 251(97.67) | 296 | 339.601±4.118 | 331.530-347.672 |  |
|  | CC | 30(63.83) | 174(67.70) | 204 | 338.735±5.190 | 328.563-348.907 | 0.628 |
|  | CT + TT | 17(36.17) | 83(32.30) | 100 | 338.010±7.031 | 324.230-351.790 |  |
| rs231775 | GG | 21(44.68) | 99(38.52) | 120 | 332.117±7.693 | 317.089-347.194 | 0.399 |
|  | AG+AA | 26(55.32) | 158(61.48) | 184 | 342.658±4.732 | 333.382-351.933 |  |
|  | AA | 8(17.02) | 32(12.45) | 40 | 335.900±11.206 | 313.936-357.864 | 0.424 |
|  | AG+ GG | 39(82.98) | 225(87.55) | 264 | 338.890±4.502 | 330.066-347.715 |  |
| rs3087243 | GG | 39(82.98) | 189(73.54) | 228 | 337.285±4.858 | 327.764-346.806 | 0.194 |
|  | AG+AA | 8(17.02) | 68(26.46) | 76 | 342.132±8.125 | 326.207-358.056 |  |
|  | AA | 2(4.26) | 14(5.45) | 16 | 331.312±22.502 | 287.209-375.416 | 0.785 |
|  | AG+ GG | 45(85.74) | 243(94.55) | 288 | 338.896±4.224 | 330.616-347.176 |  |

CI: confidence intervals, *log-rank test
